# Cost estimates of COVID-19 clinical management in Myanmar

**DOI:** 10.1186/s12913-021-07394-0

**Published:** 2021-12-27

**Authors:** Phyu Win Thant, Khin Thu Htet, Wit Ye Win, Ye Min Htwe, Thant Sin Htoo

**Affiliations:** National Health Plan Implementation Monitoring Unit, Minister’s Office, Ministry of Health and Sports, Nay Pyi Taw, Myanmar

**Keywords:** Direct costs, Indirect costs, COVID-19, Clinical management, Pandemic

## Abstract

**Objective:**

This study aims to estimate the cost of clinical management of COVID-19 infected patients based on their severity by exploring the resources used in health care provision in Myanmar.

**Methods:**

A multicenter retrospective cost analysis of COVID-19 patients was performed using the micro-costing approach from the perspective of the health system. It covered two cost components, namely direct and indirect cost of treating a patient. Input data and their quantities were obtained from COVID-19 Standard Treatment Guidelines of Ministry of Health and Sports, and administrative and financial records of resource utilization of three designated health facilities in Yangon Region. Valuation of these resources was based on the price list from the Procurement Section of the Ministry.

**Results:**

This study estimated the unit cost of clinical management of COVID-19 infected patients with *no symptom* to be *953,552 MMK(717 USD)*, with mild-moderate symptoms to be *1,155,222 MMK(869 USD)* and with severe-critically ill conditions to be *5,705,052 MMK(4290 USD)*. Average cost for a patient par day was 86,687 MMK(65 USD) for asymptomatic patients, 105,020 MMK(79 USD) for mild-moderate patients and 283,252 MMK(214 USD) for severe-critically ill patients. Since the first case detected till December 31, 2020, COVID-19 clinical management cost was accounted for 139 Billion MMK (104 Million USD) for total 124,630 confirmed cases.

**Conclusions:**

COVID-19 pandemic has caused health systems to incur the significant health care expenses. Timely implementation of the sustainable, affordable and efficient policy for COVID-19 responses is of utmost important for every nation especially in the face of a pandemic. This study provides the fundamental inputs for strategic planning, for future economic evaluations of different policy interventions, and policy recommendations for health systems to remain resilient during and after the COVID-19 pandemic in Myanmar.

## Introduction

On January 30, 2020, World Health Organization (WHO) announced the novel coronavirus outbreak as a Public Health Emergency of International Concern, later formally identified as Coronavirus Disease 2019 (COVID-19). On March 11, 2020, it was declared as a pandemic. The declaration consequently advised the member states to prepare for containment and prevention of onward spread of the virus [1]. With its advice, Myanmar has established National-Level Central Committee on Prevention, Control and Treatment of COVID-19 on March 13, 2020, for effective response to the pandemic. The virus was confirmed to have reached Myanmar on March 23, 2020. Following the detection of the first case of COVID-19, the Government of Myanmar has ramped up efforts to meet the challenges of COVID-19 by increasing testing and treatment capacity for COVID-19; providing quarantine facilities; and expanding the quantity of general and ICU beds and developing the COVID centers to isolate and treat the infected patients while safeguarding the health of other patients and health care workers. According to the World Health Organization, as of December 31, 2020, the number of COVID-19 confirmed cases reached 82.6 million, including 1.8 million deaths around the world. In Myanmar, 124,630 cases of COVID-19 confirmed cases had been reported, of which 107,069 cases were discharged from health facilities, and 2681 died. 708,370 number of suspected people were quarantined at facilities and 1,818,260 specimens were tested for COVID-19 infection between March and December, 2020 [2, 3].

Despite resource limitation, the Myanmar Government has financed the costs of the COVID-19 pandemic response that cover contact tracing, testing, quarantine and treatment of COVID-19 infected patients, by reorienting the budgetary arrangements, securing public donation and international assistance. Public hospitals and COVID Centers have been arranged to be available for admission of all COVID-19 infected patients around the country. In Yangon which is the most populated city of the country, three main health facilities have primarily been designated for caring the COVID-19 patients, namely Waibargi Specialist Hospital (90 beds), South Okkalapa Specialist Hospital (80 beds) and Phaung Gyi COVID-19 Treatment Center (1200 beds). Although there were only 374 cases and six deaths in the first wave, Myanmar has seen a dramatic rise in the number of cases in the second wave that started on August 16, 2021. Yangon became a major epicenter in the second wave and deaths became tripled with surging number of cases. To reinforce the service provision, four additional temporary facilities with a capacity of about 4050 beds in total across the Yangon Region host and treat less severe COVID-19 cases [2, 4].

Anti-pandemic measures of COVID-19 imposes disastrous resource challenges for health systems of the countries around the world [5–10]. These challenges have been substantial, especially for the low-to-middle-income countries with fewer buffering resources and poor capacity to fight against a pandemic [6, 7]. Lack of system readiness with shortage of health care workforce leads to disruption of other essential health services and self-medication. Limited health care resources with inadequate support of personal protective equipment put more at risk of health care professionals [11–14]. Poor information sharing resulted in the issue of infodemic of fake news, untrusted the Government and worsened the situation [15–17]. According to a modelling study, it is estimated that the Pandemic could cost around the United States Dollar (USD) 52 billion each 4 weeks to provide an effective health care response to COVID-19 [18].

To withstand the compiling effect of the pandemic with the minimal negative impact, health systems need to be inevitably resilient in every aspect. Considering the country’s economy and uncertainty of the pandemic duration, it alerts the policy makers to to revisit and update the policies in a timely manner according to the changing situation. Estimating the cost of treating the COVID-19 infected patients is the fundamental need that will help the clinicians and researchers as well as policy makers and health planners to handle the pandemic with competing priorities. This study aims to estimate the cost of clinical management of COVID-19 infected patients based on their severity by exploring the resources used in health care provision in Myanmar.

## Methodology

### Study design

**Fig. 1:**
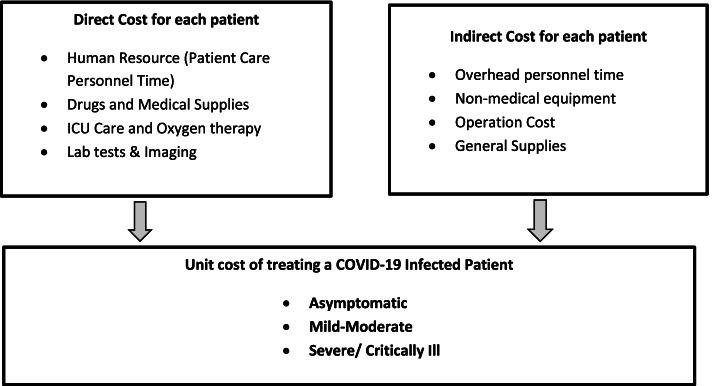
Conceptual Framework

A multicenter retrospective cost analysis of COVID-19 patients was implemented using the micro-costing approach in three health facilities from the perspective of the health system. The cost items of this exercise were mentioned in Fig. 1. It covered two cost components, namely direct and indirect costs. Direct costs were the costs that were directly attributable to patient care. It involved the cost of health workers that was spent on treating the patient, along with the entire test performed, drugs and medical supplies used including Personal Protective Equipment (PPE). The value of each of these resources (personnel time, tests, drugs, medical supplies) was identified and multiplied by the resource volume to calculate the total cost of the resources the patient directly consumed. Indirect costs, which were also called overheads, included the costs that were not directly related to the care. It covered the costs of non-medical supplies such as furniture, office materials, cost of human resources who were working in administration or management roles, and the operation costs of these facilities such as electricity, water, etc. [19] These overhead costs were estimated and allocated to the patients by averaging the cost of resources used to provide services to a patient. Patient unit costs were presented as an average cost per patient for one episode of illness and unit cost per patient day. The study was conducted by following the economic principles and collecting the detailed resource-used information, particularly the quantity and unit costs of all resources used along each individual patient’s treatment pathway in three experienced and active COVID-19 designated hospital and centers. Land and building costs were not covered in this study due to different nature of the included study sites in origin and difficult to collect the data virtually. Data on inputs and their quantities were collected from the reports of three health facilities.

### Clinical management

Department of Medical Services under the Ministry of Health and Sports (MoHS), Myanmar, has regularly updated Clinical Management Guideline for COVID-19, by being reflected by WHO and country situation. During the study period between March, 2020 and December, 2020, the most updated treatment guideline was released on August 25, 2020 and the new discharge criteria on October 10, 2020. Input data and its associated quantities for each treatment pathway were estimated based on these guidelines and criteria. According to MoHS, Myanmar, COVID-19 disease severity can be classified as follows:

1. ***Asymptomatic or Pre-symptomatic Infection***
2. ***Mild disease***
  Symptomatic patients meeting the case definition for COVID-19 without evidence of viral pneumonia or hypoxia
3. ***Moderate disease/Pneumonia***
  Adolescent or adult with clinical signs of pneumonia (fever, cough, dyspnoea, fast breathing)
4. ***Severe disease/Severe pneumonia***
  Adolescent or adult with clinical signs of pneumonia (fever, cough, dyspnea, fast breathing) plus one of the following: respiratory rate > 30 breaths/min; severe respiratory distress; or SpO2 < 93% on room air.

### Study sites

One designated hospital and two newly established centers for COVID-19 in Yangon Region, namely Waibargi Specialist Hospital, Phaunggyi COVID-19 Treatment Center and the COVID center (Thuwana), were selected for the study. Waibargi Specialist Hospital is the foremost and significant specialist hospital in Myanmar that previously treated the infectious diseases such as HIV, Multi-drug resistant TB, etc. In the COVID-19 pandemic era, it was the first designated hospital to treat the COVID-19 cases in Yangon. As Yangon is one of the densely populated cities in Myanmar, the Yangon Region Government established the Phaunggyi COVID-19 Treatment center by transforming the Central Institute of Civil Service into the Medical Center to solve the shortage of hospital beds and ICU care. It started to receive COVID-19 Inpatient in September, 2020. Private donors have contributed enormously to set up this largest COVID-19 center in Myanmar. To meet the need of the rapid surge of cases in Yangon, Thuwanna Stadium was also converted to a temporary COVID Center for treating COVID-19 patient with the main support of AYA foundation, the owner of this stadium. Inpatient care of COVID-19 cases of this center also initiated in September, 2020 [2].

### Data collection of resource use

Use of the resources for the patients was counted since the arrival at hospital/center till discharge.

### Cost estimation

A health system costing perspective was adopted and the cost per patient was measured and valued in Myanmar Kyats(MMK) and USD in the currency rate of February 1, 2021 (1 USD = 1330 MMK) [20]. Staff salaries were calculated using the average salary for each rank of staff under MoHS, apart from Intensive care unit (ICU) care of the COVID Center (Thuwanna) as it had contracted the private medical officers and nurses for ICU. In the pandemic situation, volunteers were contributing significantly to operate the COVID-19 facilities. We applied the minimum wage rate of 4800 MMK (3.6 USD) per day according to 2018 Myanmar Minimum Wage Law [21]. Unit costs of medicines, medical commodities, and medical and non-medical equipment were mostly acquired from the procurement division of MoHS. For the cost of some items that were not available from this division, the market price was used for calculation. This list comprised of the items that were procured by the facilities with their own arrangement and there was usually no financial record available. Costs of investigations and imaging were referred to the private cost in order to cover all the resources used for these investigations. For the cost of COVID-19 testing, we referred to the unpublished survey results regarding cost analysis of selected quarantine site, swab taking and lab procedures, implemented by University of Public Health, MoHS [22]. For the cost of the Convalescent Plasma, input data were received from National Blood Bank to estimate the cost for each plasma unit.

The cost of capital items including both medical and non-medical assets were estimated using the economic-based approach that covered both depreciation cost and opportunity cost of making the investment [23]. The annualization factor was calculated based on useful life and discount rate [24]. The discount rate, as recommend by WHO guide, was 3% for the base case and useful life was taken as 5 years for capital items. Annual capital costs were applied instead of using original costs as the assets were depleting on a daily basis [25, 26].

### Total cost calculation

Total estimated cost was obtained by multiplying the unit costs of clinical management and the number of cases according to the severity. For estimating the number of patients based on the disease severity, Central Epidemiology Unit of Department of Public Health and Medical Care Division of Department of Medical Services provided the necessary data [27].

### Data entry and analysis

Data on resource quantities and unit costs of the different cost components were processed and analyzed using a MS Excel spreadsheet.

## Results

### Basic characteristics of the study facilities

Table 1 describes the basic characteristics of the study facilities. Number of available beds of Waibargi Specialist Hospital was 90 beds while Phaunggyi COVID-19 Treatment center had 1200 beds and COVID Center (Thuwana) had 1000 beds. Waibargi Specialist center had accepted the first case of COVID-19 since March, 2020. The other two newly established centers had initiated their inpatient care in September, 2020. Up to December 31, 2020, Waibargi Specialist Center had treated 827 COVID-19 infected cases, Phaunggyi Center had treated 5958 cases and Thuwanna center had treated 8162 cases. Average length of stay (ALOS) for Waibargi was 20 days and ALOS for other 2 centers was around 11 days.

**Table 1:** Basic Characteristics of Study Facilities

	Waibargi Specialist Hospital	Phaunggyi COVID-19 Treatment Center	Covid Center (Thuwana)	Average No for Each facility
Number of Available Beds	90	1220	1000	*437*
Total Number of COVID-19 Patients (Up to 31st Dec, 2020)	827	5958	8162	*2262*
Total Number of ICU patients (Up to 31st Dec, 2020)	151	368	712	*173*
Average Length of Stay	20	11	11	*11*
Number of deaths due to COVID-19 (Up to 31st Dec, 2020)	65	346	184	*137*

### Unit cost of COVID-19 clinical management (asymptomatic patients)

Table 2 mentions the unit costs for treating the asymptomatic patients of COVID-19, which was composed of two types of unit – 1) per patient, and 2) per inpatient day. Unit cost for treating one asymptomatic patient was 953,552 MMK (717 USD) from the time of his/her arrival to the facility till discharged. Direct cost was accounted for 505,727 MMK (380 USD, 53% of total). Although there was no additional cost for medicine and non-COVID investigations, direct cost was mainly composed of expenses for medical commodities especially for the safety of the staff such as masks, gloves, gowns(58,985 MMK, 44 USD) and expenses for personal protective equipment (189,103 MMK, 142 USD). Total indirect cost per patient was 447,825 MMK (337 USD, 47%) which included costs for non-medical equipment (26,156 MMK, 20 USD, 3%), general staff contribution (91,186 MMK, 69 USD, 10%) and facility operation (330,483 MMK, 248 USD, 35%). Unit cost per inpatient day was 86,687 MMK (65 USD).

**Table 2:** Unit Cost of COVID-19 Case Management for Asymptomatic Patients

Inputs	Unit Cost (MMK)	Unit Cost (USD)	Percentage of Total Cost
I. Total Direct Cost per patient	**505,727**	**380**	**53%**
1. Cost for Direct Contact Health Care Personnel per patient	**48,516**	**36**	**5%**
2. Cost for Medicine and Medical Commodities	**268,699**	**202**	**28%**
2.1. Cost for Medicine per patient	–	–	0%
2.2. Cost for Medical Commodities per patient (Masks, Gloves, Gowns, Hand senstitizer, etc.)	58,985	44	6%
2.3. PPE cost per patient	189,103	142	20%
2.4. Cost of Medical Equipment per patient (BP cuff, thermometer, glucometer, pulse oximeter, syringe pump,etc)	20,610	15	2%
3. Cost of investigations and Imaging	**188,512**	**142**	**20%**
3.1. Cost for Lab investigations per patient (Non-Covid) (CP(auto), urea &eletrolytes, Liver/Renal Function tests, etc)	–	–	0%
3.2. Cost for COVID-19 Test per patient	188,512	142	20%
3.3. Cost for Imaging per patient	–	–	0%
4. Cost for Oxygen therapy per patient	**–**	–	**0%**
5. Cost for ICU care per patient (Medical Equipment)	**–**	–	**0%**
II. Total Indirect Cost per Patient	**447,825**	**337**	**47%**
1. Cost for Non-Medical Equipment per patient (Furniture, computers, generators, etc)	26,156	20	3%
2. Cost of General HR per patient (Admin staff, general workers, security, etc.)	91,186	69	10%
3. Cost of Center/Hospital Operation per patient (Electricity & Water bill, Maintenance, Meal cost, etc.)	330,483	248	35%
*Total Cost per patient* *(Direct + Indirect)*	**953,552**	**717**	**100%**
*Cost per patient per day* *(Asymptomatic)*	**86,687**	**65**	

### Unit cost of COVID-19 clinical management (mild-moderate patients)

Table 3 outlines the unit cost of COVID-19 clinical management for a patient with mild-moderate symptoms. There was a minor cost difference in the management of asymptomatic patients and patients with mild to moderate disease. The difference was due to medication for symptomatic treatment and non-COVID investigations according to the clinical guidelines. For this kind of patient, cost of facility operation(354,565 MMK, 267 USD, 31%) made up the largest share, followed by the cost of PPE(189,103 MMK, 142 USD, 16%) and COVID-19 testing (189,103 MMK, 142 USD, 16%). Average unit cost per mild-moderate patient was 1,155,222 MMK (869 USD) and average cost per patient day was 105,020 MMK(79 USD).

**Table 3:** Unit Cost of COVID-19 Case Management for Mild/Moderate Patients

Inputs	Unit Cost (MMK)	Unit Cost (USD)	Percentage of Total Cost
I. Total Direct Cost per patient	**683,315**	**514**	**59%**
1. Cost for Direct Contact Health Care Personnel per patient	**51,928**	**39**	**4%**
2. Cost for Medicine and Medical Commodities	**270,145**	**203**	**23%**
2.1. Cost for Medicine per patient	1446	1	0%
2.2. Cost for Medical Commodities per patient (Masks, Gloves, Gowns, Hand senstitizer, etc.)	58,985	44	5%
2.3. PPE cost per patient	189,103	142	16%
2.4. Cost of Medical Equipment per patient (BP cuff, thermometer, glucometer, pulse oximeter, syringe pump, etc.)	20,610	15	2%
3. Cost of investigations and Imaging	**347,512**	**261**	**30%**
3.1. Cost for Lab investigations per patient (Non-Covid) (CP(auto), urea &electrolytes, Liver/Renal Function tests, etc.)	131,000	98	11%
3.2. Cost for COVID-19 Test per patient	188,512	142	16%
3.3. Cost for Imaging per patient	28,000	21	2%
4. Cost for Oxygen therapy per patient	**13,730**	**10**	**1%**
5. Cost for ICU care per patient (Medical Equipment)	**–**	**–**	**0%**
II. Total Indirect Cost per Patient	**471,907**	**355**	**41%**
1. Cost for Non-Medical Equipment per patient (Furniture, computers, generators, etc.)	26,156	20	2%
2. Cost of General HR per patient (Admin staff, general workers, security, etc.)	91,186	69	8%
3. Cost of Center/Hospital Operation per patient (Electricity & Water bill, Maintenance, Meal cost, etc.)	354,565	267	31%
*Total Cost* *(Direct + Indirect)*	**1,155,222**	**869**	**100%**
*Cost per patient per day* *(Mild-Moderate)*	**105,020**	**79**	

### Unit cost of COVID-19 clinical management (severe-critically ill patients)

Table 4 presents the unit cost for clinical management of COVID-19 with severe-critically ill symptoms. Average unit cost per patient was 5,705,052 MMK (4290 USD) and average cost per patient day was 285,253 MMK(214 USD). Cost of medication was the main contributor of the total cost and made 24% of the total (1,347,896 MMK, 1013 USD). Other cost drivers were direct contact health care personnel cost per patient (20%, 1,161,548 MMK, 873 USD) and ICU Care (14%, 799,760MMK, 601 USD). For this type of patients, as there were the huge demand for critical care with specialized health care personals and costly medication, share of PPE cost reduced to 3% of total cost, which is 16%for mild-moderate patients and 20% for asymptomatic patients.

**Table 4:** Unit Cost of COVID-19 Case Management for Severe/Critically Ill Patients

Inputs	Unit Cost (MMK)	Unit Cost (USD)	Percentage of Total Cost
I. Total Direct Cost per patient	**5,122,004**	**3851**	**90%**
1. Cost for Direct Contact Health Care Personnel per patient	**1,161,548**	**873**	**20%**
2. Cost for Medicine and Medical Commodities	**1,616,595**	**1215**	**28%**
2.1. Cost for Medicine per patient	1,347,896	1013	24%
2.2. Cost for Medical Commodities per patient (Masks, Gloves, Gowns, Hand sensitizer, etc.)	58,985	44	1%
2.3. PPE cost per patient	189,103	142	3%
2.4. Cost of Medical Equipment per patient (BP cuff, thermometer, glucometer, pulse oximeter, syringe pump, etc.)	20,610	15	0%
3. Cost of investigations and Imaging	**837,812**	**630**	**15%**
3.1. Cost for Lab investigations per patient (Non-Covid) (CP (auto), urea & electrolytes, Liver/Renal Function tests, etc.)	621,300	467	11%
3.2. Cost for COVID-19 Test per patient	188,512	142	3%
3.3. Cost for Imaging per patient	28,000	21	0%
4. Cost for Oxygen therapy per patient	**531,004**	**399**	**9%**
5. Cost for ICU care per patient (Medical Equipment)	**799,760**	**601**	**14%**
II. Total Indirect Cost per Patient	**583,048**	**438**	**10%**
1. Cost for Non-Medical Equipment per patient (Furniture, computers, generators, etc.)	26,156	20	0%
2. Cost of General HR per patient (Admin staff, general workers, security, etc.)	91,186	69	2%
3. Cost of Center/Hospital Operation per patient (Electricity & Water bill, Maintenance, Meal cost, etc.)	465,706	350	8%
*Total Cost* *(Direct + Indirect)*	**5,705,052**	**4290**	**100%**
*Cost per patient per day* *(Severe-Critically Ill)*	**285,253**	**214**	

### Total estimated costs of clinical management of COVID-19 infected patients

On December 31, 2020, Myanmar has recorded 124,630 confirmed cases with 47,359 asymptomatic patients, 76,169 patients with mild-moderate symptoms and 1102 patients with severe-critically ill patients. Clinical management cost for these patients accounted for 139,438,249,370 MMK (104,840,789 USD) in total. It was described in Table 5.

**Table 5:** Total Estimated Cost of Clinical Management of COVID-19 Infected Patients^a^

Items	Unit Cost (MMK)	Unit Cost (USD)	No of Patients^a^	Total (MMK)	Total (USD)
1. Cost for Asymptomatic Patients	953,552	717	47,359	45,159,648,614	33,954,623
2. Cost for Mild-Moderate Patients	1,155,222	869	76,169	87,991,633,466	66,159,123
3. Cost for Severe to Critically ill Patients	5,705,052	4290	1102	6,286,967,304	4,727,043
Total Number of Confirmed Cases^a^			124,630		
*Total Cost*^a^				**139,438,249,370**	**104,840,789**

^a^Total confirmed cases and cost covered the study period from March 1st, 2020 to December 31st, 2020

## Discussion

This report presented the evidence on the costs of COVID-19 clinical management in Myanmar. Specifically, it estimated unit cost for the treatment of the COVID-19 infected patients with no symptoms, mild-moderate symptoms and severe-critically ill symptoms. The findings indicated that COVID-19 treatment costs were extensive for all three categories of patients; cost for treating severe to critically ill patients were nearly five times higher than the other two categories, accounting for 953,552 MMK(717 USD) for asymptomatic, 1,155,222 MMK(869 USD) for mild-moderate symptoms and 5,705,052 MMK(4290 USD) for severe-critically ill conditions. Treating severe-critically ill patients was extremely resource intensive to cover critical care, expensive anti-viral medication and high flow oxygen therapy. Additionally, they demanded for the huge amount of health care workers’s time including specialists such as anesthetists, ICU nurses, etc. As well-functioning ICUs were limited in the Ministry with ineffective utilization [28], and the pandemic generated the sizable resource imbalance to meet the requirement of specialized critical care health workforce. Thuwanna COVID Center solved this issue with the contracted services with the private providers for ICU care. This resulted in the direct cost for Human Resources (HR) of Thuwanna Center to be much higher than the other two facilities, and consequently the average direct HR cost became the second largest cost driver of the total cost. It highlighted that preparing ICU and strengthening the critical services, an integral part of COVID-19 crisis response, has strained significantly the health care resources, especially those of low-middle income countries with pre-existing limitations. A substantial difference between the cost of treating the different categories of the infected patients could also be found in a study of Barasa. et al. in Kenya. In their study, they mentioned that while expenses for asymptomatic patients and mild/moderate patients at health facilities were just 843.51 USD and 843.75 USD respectively, the expenses reached 1429 USD for severe patients, and 6753 USD for critical COVID-19 disease [29].

Our study also analyzed the total cost by categorizing into the direct and the indirect costs for treating the COVID-19 infected patients from health system perspective. Direct costs measured the resources used directly for services while indirect costs measured overhead use. For asymptomatic or mild-moderate patients, there was no significant difference between the percentage share of total cost between direct and indirect costs. For asymptomatic patients, direct costs were composed of two main cost items which were PPE cost(20%) and COVID-19 Testing cost(20%). Although health care workers did not need to give much time for caring asymptomatic patients, PPEs were still required for taking care of them to monitor the daily body temperature and blood pressure, to clean the space and arrange other necessities. According to Barasa. E, et.al study, Kenya health care policy applied home based care for asymptomatic or mild-moderate patients and it could be seen that costs for health care staff and PPE were much reduced for home based care, while compared to these costs for asymptomatic patients who were treated at hospitals or isolation centers [30]. For severe and critically ill patients, our study mentioned that direct costs made 90% of total cost and the overhead cost for treating them made only a small portion as care for such kind of patients required the significant workload of health care staff including specialists(1,616,595 MMK, 1215 USD per patient), expensive drugs (58,985 MMK, 44 USD per patient), PPEs(189,103 MMK, 142 USD), investigations (837,812 MMK, 630 USD), and cost for ICU care and oxygen therapy (1,303,764 MMK, 1000 USD). In Ghaffari Darab et al., study in Iran, they estimated the direct medical and indirect costs of treating the COVID-19 from a societal perspective between March and July 2020. It was found that mean cost for ICU care for a severe patient was 4384 USD, drugs and supplies cost 4313 USD, health care professional cost for 734 USD, and investigations for 572 USD [30].

In our study, we also estimated the total cost for treating all COVID-19 infected patients starting from the first case to 124,630 cases within 10-month period (March–December 2020). It was found that total 139 billion MMK (104 million USD) has been incurred during that period for treating them. This amount was equal to 3% of total current health expenditure in 2018 and 0.13% of total Myanmar GDP (2020) [30, 31]. The most updated analysis of health expenditure in Myanmar, National Health Account(2016–2018) mentioned that domestic government health expenditure in 2018 was 14% of total current health expenditure (647 billion MMK) [31, 32]. It also should be noted that our calculation of COVID care cost still did not include the treatment of comorbidities and the expenses for implementation of infection control policies of COVID-19 such as contact tracing, testing, quarantine, etc. Most importantly, it did not cover the opportunity cost of forgoing non-COVID health care that can be the big impact on budgetary space for health even if the further spread of COVID-19 infection becomes decline. Particularly, this can be caused by deferring many non-urgent health service as a consequence of COVID-19 care prioritization, disruption of essential health service provision and fear of infection of every individual [33–35].

Around the world, the COVID-19 pandemic has the significant impact on the health care budget of the countries. In china, it was found that total estimated health care cost for covid-19 was 0.62 billion USD from Jan 2020 to March 2020 [36]. Aa modelling study, published in the Lancet Global Health Journel, analyzed the cost of a COVID-19 response from the health sector of73 low income and middle income countries, and it was found that 52.45 USD billion would be incurred over 4 weeks of the pandemic and among them, 54% was for case management, 21% for maintaining essential services and 14% for rapid response and case investigations [37]. Another study in the United States by Bartsch et al. estimated that 654 billion USD would be required for direct medical cost if 80% of the US population were to get infected and 163 billion would be required if 20% were to get infected [38]. These two studies have forecasted the potential cost for health care for managing COVID-19 and highlighted the burden of the health care system and need for preparation of additional resources and financial support.

In Myanmar, Health System has already been facing many challenges due to historically institutional neglect of health sector [39]. Although both public and private sectors are in a pluralistic mix in health care market, MoHS plays the major role to cover health service provision throughout the country. According to Ministry Data Information System, there are 1177 public hospitals under MoHS, categorizing into general hospitals, specialist and teaching hospitals, regional/state hospitals and district hospitals, township and station hospitals. In rural areas, rural health centers and sub-rural health centers to provide health services, including public health services [40]. Despite the fact that there is free health care policy, lack of clearly defined eligibility and system readiness makes patients to pay out of pocket payment at the point of care. Health insurance and social protection in Myanmar are negligible and there is relatively low financial protection mechanism for receiving health care services [41]. With these limitations, when the pandemic affects the country, the Government of Myanmar attempted to give care to all infected patients regardless of the severity of symptoms and affordability without taking any charge, providing all supplies including accommodation, food and medicines. Private sectors were not allowed to test or treat COVID-19 suspected patients till the end of the study period. To bridge the financial gap in encountering the pandemic, it has been complemented by diverting several budgetary sources including the government supplementary budget, general reserve fund from reallocation of line ministries [42], public donation and international aids.

As the timeline for COVID-19 Pandemic cannot be predicted, financial sustainability becomes the priority matter for the Government to ensure the continuity of care and not to transfer the financial burden to the people by paying out of pocket health care expenses. To address this issue, Myanmar policy makers are in urgent need to find the ways to face an unexpected demand surge of the pandemic in more effective and efficient ways. It should be considered to engage with private health care providers by easing regulations governing facility closure and purchasing the needed health services with certain form of payment to ensure sufficient access to, and utilization of health care services during the COVID-19 Pandemic. Additionally, home-based care for COVID-19 infected persons without symptoms should be taken into consideration as this can prevent over workload of limited health centers and increase accessibility to the non-COVID essential health service provision. Innovative digital solutions including tele consultation is another option to prevent the further spread of COVID-19 and promote access to the health services. Better preparation for early detection and rapid response will mitigate the future costs of COVID care by reducing infection rate. For the long run, health financing reform should be arranged systematically to raise health revenue, to reduce inefficiencies and purchase the health services strategically.

### Strengths and limitation

This study had a number of strengths. As it applied a bottom-up micro-costing, it involved the detail identification and quantification of resources that were used in clinical management of COVID-19 since admission till discharge. In addition, it covered three health facilities with different nature, composing one public designated hospital and two public-private temporary facilities. This allowed potential for generalization across wider healthcare economies. There were some limitations of this study that should be recognized. This study is based on normative costing based on Standard Treatment Guidelines of Ministry to identify and measure the resources used. However, in reality, there may be some variations in practices across the different hospitals and across the countries. Further studies are recommended to get the costs of the health facilities at State/Region level and Township level to get the complete picture of financial implications of different policy options. Although there may be potential accuracy issues of the data presented in this study due to the time constraint and pandemic situation, an extensive effort was made to identify all relevant data about each designated health centers, capture all valid cost items and ensure the validity. In spite of these limitations, it should be highlighted that this is the initial effort to point out the clinical management cost of COVID-19 in Myanmar.

## Conclusion

COVID-19 Pandemic has caused health systems to incur the devastating expenses of health care service provision. Timely formulation and implementation of the sustainable, affordable and efficient policy for COVID-19 responses is of utmost important for every nation especially in face of the pandemic. It is the crucial stage for the Myanmar policy makers to prepare the private sector engagement, innovative digital interventions and revisit the admission policy and inevitably health financing reform to mitigate the economic consequences of COVID-19 case management. This study provides the fundamental inputs for strategic planning, for future economic evaluations of different policy interventions, and for policy recommendations to remain resilient during and after the COVID-19 pandemic in Myanmar.

## Data Availability

The datasets analyzed during the current study are available from the corresponding author on reasonable request.

## Abbreviations

WHO: World Health Organization
MoHS: Ministry of Health and Sports
COVID-19: Coronavirus Disease 2019
ALOS: Average length of stay
ICU: Intensive care unit
USD: The United States dollar
